# ThyPRO-IR: Translation and Linguistic Validation of the Persian Version of Thyroid-Specific Quality of Life (QoL) Patient-Reported Outcome (PRO) Questionnaire for Benign Thyroid Disorders

**DOI:** 10.5812/ijem-149014

**Published:** 2024-07-29

**Authors:** Zohreh Maghsoomi, Mohammad Ebrahim Khamseh, Mojtaba Malek, Afsaneh Dehnad, Shirin Mohamadzadeh, Torquil Watt, Ramin Malboosbaf

**Affiliations:** 1 Research Center for Prevention of Cardiovascular Disease, Institute of Endocrinology and Metabolism, Iran University of Medical Sciences (IUMS), Tehran, Iran; 2 Endocrine Research Center, Institute of Endocrinology and Metabolism, Iran University of Medical Sciences (IUMS), Tehran, Iran; 3 Department of Medical Education, Center for Educational Research in Medical Sciences (CERMS), School of Medicine, Iran University of Medical Sciences (IUMS), Tehran, Iran; 4 Department of English Languages, Alzahra University, Tehran, Iran; 5 Department of Medical Endocrinology, Hospital Rigshospitalet, Copenhagen University, Copenhagen, Denmark

**Keywords:** ThyPRO-39, ThyPRO-IR, Questionnaire, Quality of Life, Thyroid Disorders

## Abstract

**Background:**

Considering the high prevalence of benign thyroid disorders, the availability of an instrument measuring health-related quality of life (HRQoL) in this population is very important.

**Objectives:**

The current study aims to translate and validate the Persian version of the ThyPRO-39.

**Methods:**

In accordance with standard methodology, a double forward, reconciliation, and backward translation of the questionnaire was conducted. A field consultant identified discrepancies between the original questionnaire and the back translation. Discrepancies were addressed, revised, and retested before submitting it for developer review. Finally, five cognitive interviews were conducted among patients with benign thyroid problems to ensure alignment between their understanding of the Persian items and their original English counterparts.

**Results:**

Translation and linguistic validations of the Persian version of the ThyPRO-39 Questionnaire were developed according to the established rules. Two translators did the forward translation with no significant disagreement. Considering backward translation, the field consultant changed eight items, and the developer provided seven additional comments. After interviewing five patients, nine revisions were performed by the field consultant. Finally, an external consultant reviewed all changes and approved the questionnaire.

**Conclusions:**

We translated and linguistically validated the Persian version of the ThyPRO-39. Now, the ThyPRO-IR is ready for assessment of thyroid-specific QoL in Iranian patients with benign thyroid disorders.

## 1. Background

After diabetes mellitus, benign thyroid disease has been known as the second most common endocrine problem worldwide (1). Impaired quality of life (QoL) despite standard treatments has been identified in numerous studies (2, 3). Therefore, a comprehensive questionnaire is needed to assess these patients' health-related quality of life (HRQoL) (4). Different studies have been performed for QoL assessment in benign thyroid diseases, which refer to a group of thyroid disorders that affect the function and structure of the thyroid gland, such as autoimmune and nonautoimmune thyroid hypo or hyperfunction and goiter. They all highlight the importance of the review of QoL beyond the laboratory test evaluation (5, 6).

### 1.1. ThyPRO: A Thyroid-Specific Quality of Life Questionnaire in Patients with Benign Thyroid Disease

In recent decades, many studies have dealt with HRQoL in patients with benign thyroid disease (5). Thyroid–Specific Patient Reported Outcome (ThyPRO) is widely applied in benign thyroid disease studies and has clinical validity and reliability, which led to translation into 23 languages with high intercultural validity (1, 4, 5, 7-13). Therefore, ThyPRO was chosen for translation into Persian among all the questionnaires. Since ThyPRO's original version, which contains 85 items, is relatively long in the clinical setting (taking approximately 15 minutes), a shorter version of 39 items taking only 5 minutes to complete was created in 2015 and is now being recommended for clinical use (14). The ThyPRO-39 (Table 1) is summarized in 13 single-construct and one overall summary scales: Hyper- and hypothyroid symptoms, goiter symptoms, eye symptoms, tiredness, cognitive problems, anxiety, depression, emotional susceptibility, impact on social and daily life, cosmetic complaints, impact on overall HRQoL and the Composite Scale, summarizing the well-being, function, and impact scales in one score. The patient rated each question based on the Likert Scale: Not at all, a little, some, quite a bit, very much.

**Table 1.** The List of 13 Multi-Item Scales of ThyPRO-IR

Variables	Symptoms
**Goiter symptoms**	
1a	A sensation of fullness in the neck
1C	Pressure in your throat
1h	Discomfort swallowing
**Tiredness**	
2a	Been tired
2c	Difficulty getting motivated
3b	Energetic
**Emotional susceptibility**	
7c	Easily felt stressed
7d	Mood swings
7h	Felt in control of life
**Hyperthyroid symptoms**	
1l	Trembling hands
1m	Tendency to sweat
1n	Palpitations
1t	Upset stomach
**Cognitive complaints**	
4a	Difficulty remembering
4b	Slow or unclear thinking
4f	Difficulty concentrating
**Impaired social life**	
8a	Difficulty being with other people
8b	A burden to other people
8c	Conflicts with other people
**Hypothyroid symptoms**	
1q	Sensitive to cold
1cc	Swollen hands or feet
1dd	Dry skin
1ee	Itchy skin
**Anxiety**	
5b	Afraid or anxious
5c	Felt tension
5e	Uneasy
**Impaired daily life**	
9a	Difficulty managing daily life
9c	Not being able to participate in life
9e	Everything takes longer to do
**Eye symptoms**	
1w	Grittiness in eyes
1x	Impaired vision
1bb	Very sensitive to light
**Depression symptoms**	
6a	Sad
6e	Unhappy
6g	Self-confident
**Cosmetic complaints**	
11a	Thyroid disease affects the appearance
11d	Bothered by other people looking
11e	Influence on clothes worn
**Overall quality of life**	
12	Thyroid disease has a negative effect on the quality of life

All scales (except for the hypothyroid symptoms, the Overall QoL Scale, and the Composite Scale) are transformed to range 0 - 100 according to Table 2. 

**Table 2.** Scoring Symptoms ^a^

Raw Sum Score	Final Rescaled Short-Form Score										
	Goiter	Hyperthyroid	Eye	Tiredness	Cognition	Anxiety	Depression	Susceptibility	Social Life	Daily Life	Appearance
**0**	2	2	1	0	1	1	0	1	0	0	1
**1**	10	8	8	8	7	10	7	7	8	7	12
**2**	15	13	14	17	14	18	14	13	17	15	21
**3**	20	18	20	25	21	26	22	21	25	22	28
**4**	26	23	25	33	29	34	29	28	33	30	36
**5**	31	28	32	42	37	41	37	36	42	38	43
**6**	37	33	38	50	44	49	45	44	50	46	51
**7**	43	38	45	58	52	56	54	52	58	54	59
**8**	49	44	52	67	60	63	63	60	67	62	66
**9**	57	49	60	75	68	71	71	68	75	71	73
**10**	64	55	68	83	76	79	80	77	83	80	80
**11**	73	60	78	92	85	87	89	86	92	89	87
**12**	84	66	89	100	95	96	97	95	100	98	96
**13**	-	71	-	-	-	-	-	-	-	-	-
**14**	-	77	-	-	-	-	-	-	-	-	-
**15**	-	84	-	-	-	-	-	-	-	-	-
**16**	-	90	-	-	-	-	-	-	-	-	-

^a^ The raw score can be found on the left. Each scale has a 0 - 100 score that is tabulated separately. For instance, a patient who scores 6 on the Tiredness scale would have a Tiredness symptom score of 50 out of 100 if they answered "some" to all three Tiredness items.

## 2. Objectives

The current study aimed to translate and linguistically validate the ThyPRO-39 Questionnaire into Persian.

## 3. Methods

First, two native Persian translators who were fluent in English independently translated the original version of the questionnaire into Persian (forward translation). Then the two versions were compared and agreed on all items (reconciled version). The reason for selecting any item was well documented, including discrepancies between the two forward translations. In the next step, the forward-translated version was back-translated by a native English speaker fluent in Persian (backward translation). A field expert familiar with patient-reported outcomes (PROs) research and fluent in both Persian and English reviewed the backward-translated version, identified any discrepancies, and suggested how these should be handled. These comments were considered in the back-translated version. The questionnaire developer (TW) reviewed and confirmed the back-translated version. Then, the Persian wording was entered into the questionnaire format (based on the U.S. version). Five patients with benign thyroid problems were tested using the cognitive interview method to evaluate their understanding of the questionnaire. Any changes for better patient understanding were made and documented under the observation of one expert. An external field expert reviewed all changes. All comments/changes were recorded.

## 4. Results

Translation and linguistic validations of the Persian version of the ThyPRO-39 Questionnaire were developed according to the established rules. Two translators did the forward translation with no significant disagreement. Considering backward translation, the field consultant changed eight items, and the developer provided seven additional comments. After interviewing three hypothyroid and two hyperthyroid patients, which took about 12 minutes for each interview, nine revisions were performed by the field consultant. Finally, an external consultant reviewed all changes and approved the questionnaire (Table 3). 

**Table 3.** Review One Item as a Part of the Translation Process

The Translation Process of Item 7d	Results
**Original ThyPRO**	Had trembling hands?
**Wordings by the first forward translator**	Hand tremors?
**Wordings by the second forward translator**	Did you have hand tremors?
**Reconciled Persian translation**	Did you have hand tremors?
**Wordings by the back translator**	Had tremor in your hands?
**The consultant's comments**	Not considered important.
**The developer's comments**	Does the current Persian version reflect the most natural way of expressing this in Persian?
**Response to the developer's comments by the consultant**	We appreciate your comment. This expression is current in the Persian version.
**The result of changes included in ThyPRO-IR**	Did you have hand tremors?

## 5. Discussion

In this study, we performed a translation and linguistic validation of the Persian version of the ThyPRO-39 Questionnaire. The original and other translated versions have been widely used in various studies to assess the quality of life of thyroid-specific patients.

-The importance of measuring the quality of life of patients with benign thyroid disorders: Although benign thyroid disorders are not life-threatening, they play an essential role in the quality of life and well-being of patients. Quality of life assessment could help clinicians reach comprehensive care beyond treating physical symptoms. Additionally, considering PROs in clinical practice could ensure our treatment is compatible with patients' preferences, which demonstrates a Patient-Centered Approach (2). Moreover, quality of life assessment could help physicians evaluate treatment outcomes, such as thyroid surgery outcomes in patients with goiter (15).

-Using ThyPRO-39 in patients with benign thyroid disease: In patients with benign thyroid disease, the ThyPRO instrument was found to be superior to other quality-of-life questionnaires. The measurement properties assessed by six studies revealed that ThyPRO has "strong adequate evidence for internal consistency, content validity, and structural validity and moderate adequate evidence in hypothesis testing and cross-cultural validity" (9).

ThyPRO-39 can be utilized in clinical settings to give valuable insights into the patient's viewpoint on their condition and treatment. On the other hand, the instrument's responsiveness to changes in patients' conditions over time makes it a helpful questionnaire for both clinical practice and research (16). For instance, in patients who have just been diagnosed with Graves' disease and treated with antithyroid medications, the ThyPRO-39 questionnaire was utilized to monitor changes in HRQoL throughout one year. According to the study, several scales of the questionnaire, including hyperthyroid symptoms and anxiety, improved significantly after 6 and 12 months of treatment (17). In future clinical trials of combination thyroid hormone therapies for hypothyroid conditions, it has been suggested that ThyPRO-39 be used as a reliable, valid assessment that is easily applicable (18).

Recent research on applying PROs as routine practices has revealed that medical professionals and investigators frequently encounter practical difficulties, including administrative processes and interpretation. Thus, the abovementioned points need to be considered before using ThyPRO in clinical practice (6). Moreover, the ThyPRO-39 is a thyroid-specific questionnaire and, therefore, may not adequately address comorbid conditions that could also affect the patient's quality of life (16).

Considering the abovementioned, the ThyPRO-IR could help clinicians have a patient-centered approach and the ability to monitor their patients in terms of well-being and daily functioning even after long-term treatment beyond improving physical and laboratory findings. Moreover, it could help Iranian researchers develop their research goals based on patient-reported outcomes.

### 5.1. Conclusions

We translated and linguistically validated the Persian version of the ThyPRO-39. Now, the ThyPRO-IR is ready for assessment of thyroid-specific QoL in Iranian patients with benign thyroid disorders.

## Data Availability

The dataset presented in the study is available on request from the corresponding author during submission or after publication.
